# Oral health education program among pre-school children: an application of health-promoting schools approach

**DOI:** 10.15171/hpp.2016.26

**Published:** 2016-08-10

**Authors:** Mahboube Shirzad, Mohammad Hossein Taghdisi, Tahereh Dehdari, Jamileh Abolghasemi

**Affiliations:** ^1^Department of Health Education and Health Promotion, Iran University of Medical Sciences, Tehran, Iran; ^2^Department of Biostatistics, Iran University of Medical Sciences, Tehran, Iran

**Keywords:** Oral health, Pre-school children, Albanian’s Health Promoting Schools Model

## Abstract

**Background:** Preschool children have a limit ability to take care of their teeth. The aim of this study was to determine the effect of an intervention based on Albanian’s Health Promoting Schools Model (Albanian’s HPSM) on the oral health behaviors among a group of Iranian female preschool (5-6 years old) children.

**Methods:** In this quasi-experimental study, 120 children in seventh district of Tehran, Iran were randomly recruited and assigned to either the intervention or the control groups. A scale was designed and validated to assess the oral health behaviors among the children and knowledge,attitude, self-efficacy beliefs, perceived barriers and oral health behaviors among the parents and the schoolteachers. An expert panel approved the content validity of the scale (CVR = 0.89,CVI = 0.90). The reliability was also approved applying intraclass correlation coefficient (range,0.83–0.92) and Cronbach alpha (range, 0.83–0.96). Based on the preliminary data, a 6-week intervention was designed and conducted to the intervention group. One month following the intervention, both groups were followed-up. The data were analyzed using covariance and paired t tests.

**Results:** Following the intervention, significant differences were found in the oral health behaviors of the children in the intervention group (P < 0.05) and knowledge, attitude, oral health behaviors, self-efficacy, and perceived barriers of their parents and the schoolteachers (P < 0.05).

**Conclusion:** Using Albanian’s health-promoting schools (HPSs) approach was useful in improving the oral hygiene behaviors among the preschool children.

## Introduction

Pre-school children are at high risk for dental caries.^1^ About 51.7% of Iranian children, aged 3 to 5 years, have tooth decay and further efforts are essential to achieve 90% caries-free teeth among 5-year-old children.^2^ Various factors have been identified to affect children’s teeth decay including poor oral hygiene and nutritional status among the children as well as the level of oral health-related knowledge, habits, attitude and self-efficacy among the schoolteachers and parents.^3-9^ Such variables should be considered when developing oral health education programs targeting preschool children.

Oral health education can be reinforced throughout the school years, an influential period in children’s lives. During school years lifelong beliefs, positive attitudes and personal skills among the children are being developed.^10^ As noted by Kwan et al, oral health education should form part of all subjects in the school curriculum and involve students, school staffs and parents in health promotion activities at school.^10^ Oral health education should be regularly reinforced at home by health-promoting school (HPS) programs, and should be also developed at key educational stages throughout the children’s school career.^11^

The effectiveness of interventions adopting a HPSs approach are likely to be increased.^12^ The schools that constantly strengthen their capacity as a healthy setting for living, learning and working are considered as HPSs.^13^ HPSs enable students to take control over their health and become the future active and responsible citizens in their society.^14^ Such schools help to involve the school and community members in planning the programs addressing their health needs and can be maintained and sustained with available resources and commitments.^15^ Previous literature have shown that adopting HPS approaches to develop nutrition promotion programmes increased the intake of high-fibre foods, healthier snacks, water, milk, fruit and vegetables and performing the oral health practices among students.^16-18^ It can also reduce ‘breakfast skipping,’ consumption of red food, low-nutrient dense foods, fatty and cream foods, sweet drinks consumption, eating disorders and smoking among students.^17-19^

**Figure 1:**
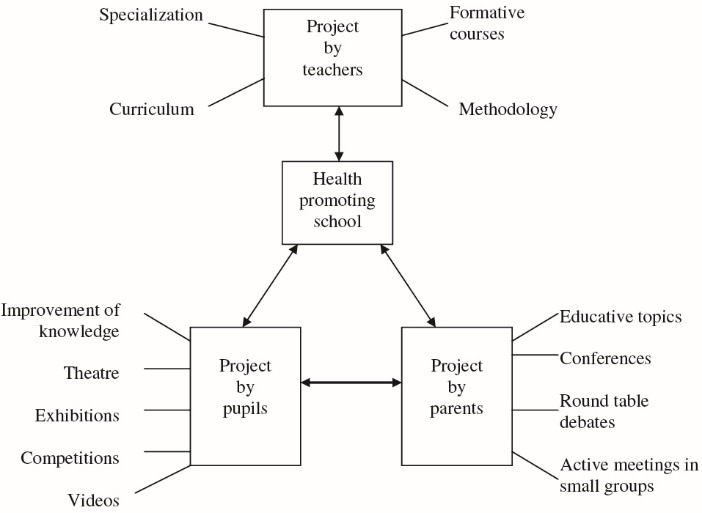
Albanian’s Health Promoting Schools Model.^20^

Albanian^’^s Health Promoting Schools Model (Albanian^’^s HPSM), as one of the HPSs approaches, was developed in the field of health education for primary schools. This model consists three basic branches including pupils, teachers and parents and emphasizes good relationships and proper collaboration between these branches.^20^ In Albanian^’^s HPSM (Figure 1), the educational methods for training pupils (e.g. videos, health competitions and theatre), parents (e.g. meetings and small groups) and schoolteachers (e.g. formative courses and seminars) have been described.^20^ Although Albanian^’^s HPSM is recommended for developing educational interventions in primary schools,^20^ few researches have studied this model to promote healthy behaviour among preschool children.^21,22^

Considering the high prevalence of tooth decay among Iranian children^23,24^ and the effectiveness of adopting a HPSs approach to develop educational intervention on the oral health of children^16,19^ and, also, the lack of intervention studies in this field, this study was conducted to determine the effect of an educational intervention based on Albanian^’^s HPSM on oral health behaviors among a sample of pre-school (5-6 years old) children in Tehran, Iran.

## Materials and Methods

### Participants and setting

This quasi-experimental study was conducted from April to September 2015. Among the 7 middle-income areas of Tehran, the area number 7 was randomly selected, from which four preschool centres were, also, randomly selected. Preschool children in two schools were assigned into the intervention group and those in the other two schools were considered as the control group. Then, according to the estimated sample size, 30 preschool (5-6 years old) children were randomly recruited from each school. Inclusion criteria in the study were the student’s agreement to participate in the study, ability to read and write Persian, residency in the city of Tehran and being in the preschool grade. The schoolteachers and parents of the students were participated in the study. None of the students and their parents and schoolteachers refused to take part in the study. Finally, 60 students, 60 parents (1 parent of every student) and 11 schoolteachers were included in each group. Demographic characteristics of the children in the two groups are presented in Table 1. At baseline, no significant differences were observed between the two groups in the demographic characteristics.

### Sample size calculation

In this study, M (the number of clusters) = 18, V_2 (_the estimated variance on oral health behavior among school children in a study by Okada et al^6^) = 0.123, ε (the margin of errors) = 0.01, α = 0.05 and β = 0.20. To calculate the sample size, the formula (n= [(z_1-α/2_ +z_1-β_)^2^MV_1y_^2^/[(z_1-α/2_ +z_1-β_)2(M-1*)* ε^2^) was used. The final sample size was 120 participants with 60 in each group (control and intervention).

### Study instruments and measures

The students’ information on oral health behaviors, as well as their parents^’^ and schoolteachers^’^ self-efficacy beliefs, perceived barriers, knowledge, attitude and oral health behaviors were collected using a self-administered questionnaire developed by the researchers. In order to develop the instruments, a literature review was done and 20 female pre-school children and their schoolteachers and parents were interviewed to collect their opinions concerning oral health. Initial instruments were generated and consequently qualitative face and quantitative content validity of the items were evaluated. Thirty female students and their parents and schoolteachers were asked to comment on the simplicity, readability and clarity of the items. According to their opinions, several questions were deleted. For calculating the content validity, an expert panel including ten specialists in the areas of health education and dentistry reviewed the necessity and the relevance of items.

The necessity of the items was assessed using a 3-point rating scale: E indicated essential; U, useful but not essential; and N, not necessary. The relevance of the items was also assessed using a 4-point rating scale: (N) not relevant, (S) slightly relevant, (R) relevant, and (V) completely relevant. Based on the experts’ opinions, the content validity index (CVI) and content validity ratio (CVR) of each item were assessed. Items having CVR less than 0.62 and CVI less than 0.78 were deleted.^25,26^ In the present study, the CVI and CVR of the scales, as a whole, was 0.90 and 0.89, respectively.

To estimate the reliability of the scales, intraclass correlation coefficient (ICC) and Cronbach alpha procedures were used with 20 female children and their parent and schoolteachers (with a 2-week interval between each test). The satisfactory value for the ICC and Cronbach alpha was considered ≥ 0.40 and ≥0.70, respectively.^27,28^

#### Knowledge of parents and teachers about oral health

One question (‘in your opinion, which of the following factors affect oral health?’) with 14 items on a 3-point scale (0=No, 1=I don’t know, 2=Yes) were used to measure the knowledge of parents and teachers regarding oral health. Cronbach alpha for the knowledge scale was 0.83. The ICC for this scale was 0.78.

#### Attitude of parents and teachers toward oral health

A nine items scale was used to measure the attitude (e.g. ‘Brushing makes me feel good’). The items in this scale were measured based on a Likert-type scale ranging from 1=‘strongly disagree’ to 5=‘strongly agree’. Cronbach alpha for this scale was 0.87 and the ICC was 0.79.

#### Oral health behaviors in teachers and parents

Fourteen items constituted the oral health behaviors scale (e.g. ‘Do you brush your teeth every night before going to bed?’). The items of this scale were measured based on a Likert-type scale ranging from 1=“never” to 4=“always”’. Cronbach alpha estimated for this scale was 0.92 and the ICC was 0.93.

#### Perceived self-efficacy of parents and teachers regarding oral health behaviors

A 10 items scale was designed to measure the parents and teachers ^‘^s self-efficacy beliefs to adopt oral health behaviors (e.g. ‘At nights, although I am too tired, I brush my teeth before going to bed’). The items in this scale were measured on a Likert-type scale, ranging from 1=‘completely unconfident’ to 5=‘completely confident’. Cronbach alpha of this sub-scale was 0.75 and the ICC was 0.95.

#### Perceived barriers for adopting oral health behaviors in parents and teachers

Ten items were designed to measure the perceived barriers (e.g. ‘I feel nausea after using mouthwash’). These items were measured on a Likert-type scale ranging from 1=‘strongly disagree’ to 5=‘strongly agree’. Cronbach alpha of this scale was 0.84 and the ICC was 0.81.

#### Oral health behaviors in preschool children

Eight items on a 2-point scale (0=No, 1=Yes) were designed to measure the oral health behaviors among children (e.g. ‘Do you brush your teeth every night before going to bed?’). Cronbach alpha estimated for this scale was 0.96 and the ICC was 0.85.

#### Intervention program

Based on the primary diagnostic assessment, an educational intervention was designed and performed for the children their parents and schoolteachers in the intervention group.

#### Manipulation program on children

Six 45-minutes training sessions for the children were held. In the first session, after presenting a story regarding oral health, the children were encouraged to discuss their positive and negative beliefs about the oral health. In the second session, children drew paintings regarding the oral health and, by posing some open-ended questions, the students were asked to share their experiences and feelings with other participants upon their paintings. In the third session, some oral health games and entertainments such as solving puzzles, connecting points and so on were presented. In the fourth session, all children read a poem together about the oral health. In the fifth session, three films and animations about the essential behaviors to maintain good oral health were shown. Finally, in the sixth session, the correct methods of tooth brushing and flossing were demonstrated to the children.

#### Manipulation program on parents and schoolteachers

Four 45-minutes sessions for the parents and schoolteachers were held. In the first educational session for the parents and schoolteachers, the importance of preschool-aged children’s oral health and the necessity of taking care of the milk teeth among children were discussed. In this meeting, they were encouraged to pay more attention to the status of oral health and to persuade children towards good performance in oral health behaviors. In the second session, a lecture was presented on the prevention strategies of tooth decay and their role in maintaining good oral health in children. In the third and fourth sessions, by posing some open-ended questions, the parents and schoolteachers were asked to discuss about the positive and negative beliefs and experiences regarding the oral and dental health. In these sessions, through verbal persuasions, they were assured to be able to reduce the barriers in performing oral health behavior by the children. The parents in the intervention group were also given a booklet about the importance of oral health in preschool children, the benefits of tooth decay preventive behaviors, and the ways to overcome the barriers to adopt oral health behaviors among children.

One month after the intervention, the questionnaires were delivered to the two groups and all completed the questionnaires again.

#### Statistical analyses

The data were analysed using the SPSS statistical software (version 16). The normality of the data was examined by Kolmogorov–Smirnov test. The homogeneity of demographic characteristics of the two groups at baseline was analysed by chi-square and Fisher exact tests. Also, paired *t* tests were used to test the within-group changes. Differences in the outcomes between the two groups before and after the intervention were tested using the independent-samples *t* test and analysis of covariance (ANCOVA), respectively. The data were expressed as mean (standard deviation [SD]). Significance of all the results was considered as *P*<0.05 level, at baseline.

## Results

### Outcomes for the schoolteachers and parents of children

Mean scores of attitude, knowledge, perceived self-efficacy, and adopting oral health behaviors for both groups before and after intervention are shown in Table 2. The results of the paired samples *t* test showed a significant increase in self-efficacy beliefs, knowledge, attitude and adopting oral health behaviors scores of the schoolteachers and parents of children in the intervention group after the intervention compared to the baseline. In addition, a significant reduction was observed in perceived barriers score of this group compared to the primary data. Results also indicated that after the intervention the parents and schoolteachers of the children reported a significant increase in perceived self-efficacy, knowledge, attitude and adopting oral health behaviors compared to the parents and schoolteachers of children in the control group (Table 2). In addition, there were significant reductions in perceived barriers of parents and schoolteachers of children in the intervention group compared with the parents and schoolteachers of children in the control group (Table 2).

### Behavioral outcome for the children

Findings showed that the educational intervention had significant effect on the mean score of oral health behaviors of children in the intervention group compared to the control group (Table 2).

## Discussion

Results of the study showed that the Albanian^’^s HPSM-based intervention considerably increased adopting oral health behaviors among the preschool children in the intervention group compared to those in the control group. This finding is similar to those found in the previous studies which concluded that conducting health promoting school programs can reduce various health problems such as smoking, low consumption of fruits and water and inadequate oral health behaviors among the students.^16-18^ In consistent with previous studies it can be claimed that trained teachers and parents play an important role in encouraging students to adopt a sustainable healthy lifestyle for good oral health.^15,29-32^ Although the teachers and parents’ oral health behaviour, as role models, may influence the children’s gingival health and dental caries,^6^ it has been shown that many of these role models have limited knowledge and awareness about the oral health.^15^ Therefore, these groups need training and school is an ideal setting that can provide a participatory environment to work with them to promote the children’s oral health.

In this study, after the intervention, the teachers and parents of the intervention group had higher self-efficacy belief scores towards oral health behaviors than their counterparts in the control group. Some previous studies emphasized the role of maternal oral health self-efficacy in children’s oral hygiene.^5,32^ As perceived self-efficacy has been recognized as one of the important predictors for adopting oral hygiene behaviors, developing programs aimed at fostering mothers^’^ self-efficacy may promote the healthy dental habits among children. The negative association between self-efficacy beliefs and perceived barriers has been shown in literature.^33,34^ The higher self-efficacy belief results in fewer perceived barriers in performing a target behavior.^33-35^ In line with these findings, in present study it was found that perceived barriers of parents and teachers for adopting oral health practice significantly decreased after the intervention compared to the parents and teachers of children in the control group. In the present study, the perceived barriers had a negative correlation with perceived self-efficacy for oral health behaviors. Several barriers for adopting oral health behaviors in children and their parents have been noted in a previous study.^36^ Focus on addressing the anticipated barriers for adopting oral health behaviors through intervention efforts may be considered as a good strategy to increase self-efficacy beliefs among parents and teachers of preschool children, as those conducted in the present study.

Similar with those found by Rong et al,^32^ following the intervention, significant differences were found in the mean scores of knowledge, attitude and adopting oral health behaviors among the parents and teachers of children in the intervention group compared to their counterparts in the control group. Several studies have confirmed the need to enhance the knowledge and modify the attitude of teachers and parents regarding the oral hygiene.^6,37-40^ As these cognitive factors are potentially modifiable,^5^ providing educational programs aimed at addressing these factors in schools could be effective in increasing the oral health literacy among teachers, parents and establishing dental hygiene habits among their children.

***Strengths and limitations***

Although the study highlights the application of Albanian^’^s HPSM framework to develop an oral health education intervention, there are some limitations; firstly, the data were collected from a small sample of Iranian female preschool children in middle-income areas of Tehran, Iran which posed a constraint on the generalizability of the findings. Secondly, male children were not included in the study. Thirdly, the homogeneity of the sample may limit the generalizability of the findings to the other preschool children residing in other areas of Tehran. As a final limitation for the present study, the short duration of the follow-up sessions can be noted. This was due to the time limitations of the researchers.

### Implications for policy and practice 

Health policymakers should consider such studies applying HPSs approach to provide more evidence based policies and to build and extend the capacity of the schools in promoting the oral health of the preschool children. Practitioners, school nurses and school health workers should pay a more specific attention to the use of health-promoting approaches for developing oral health promotion interventions in the schools.

## Conclusion

In conclusion, several benefits may be obtained from adopting a HPS approach to develop oral health education interventions. Schools can provide a participatory and supportive environment in order to involve teachers and parents in the process of oral health promotion of the preschool children.

## Acknowledgements

This MSc thesis was funded by Iran University of Medical Sciences grant number 94-01-27-25825.

## Ethical approval

The study was approved by the ethics committee in Iran University of Medical Sciences (code# 94-01-27-25825). Also, the present study protocol was registered in the Iranian registry of Clinical Trials Center (code# IRCT201504207352N10). Children, their parents and schoolteachers were informed about the objectives of the study and a written consent was obtained from them.

## Competing interests

None of the authors have any conflict of interest.

## Authors’ contributions

MS and MHT initiated and designed the study. With the help of TD, GA and MS designed and validated the scale. MS gathered the data. Data analysis of pre- and post-tests was done by GA and MS. TD, MS and MT designed and conducted the educational sessions. All co-authors made substantial contributions to data analysis and interpretation and writing of the manuscript.

**Table 1:** Demographic characteristics of the children in the two groups

	**Control group**				**Intervention group**				***P***
	**N**	**%**	**Mean**	**SD**	**N**	**%**	**Mean**	**SD**	
Age of the parents			36.9	6.54			34.9	5.09	0.135
Age of the schoolteachers			37.2	1.69			36.7	1.92	0.455
Mother’s education level									
<12th grade	21	36			25	44			0.560
>12th grade	37	64			32	56			
Father’s education level									
<12th grade	29	50			31	54			0.457
>12th grade	28	40			26	46			
Family income per month									
< US$300	10	19			9	16			
US$300-600	32	60			34	62			0.120
> US$600	11	21			12	22			
Occupation of mother									
Employee	13	22			13	23			0.134
Housewife	40	70			38	67			
Other	5	6			6	10			
Occupation of father									
Employee	20	36			25	45			0.337
Self-Employment	29	52			28	50			
Other	7	12			3	5			

**Table 2:** Comparison of the mean scores of the Albanian^’^s HPSM constructs and oral health behaviors scale in the student and their parents and schoolteachers before and after the educational intervention

	**Control group**			**Intervention group**				
	**Before intervention Mean (SD)**	**After intervention Mean (SD)**	***P*** ****** ^a^	**Before intervention** **Mean (SD)**	**After intervention Mean (SD)**	***P*** ****** ^b^	***P*** **** ^c^	***P*** ****** ^a^
**Parents**								
Knowledge	44.17 ± 3.29	44.19 ± 3.29	0.931	44.23 ± 5.65	46.49 ± 3.88	0.001	0.940	0.006
Attitude	42.81 ± 29.69	43.20 ± 3.11	0.374	42.66 ± 3.34	44.13 ± 1.26	0.011	0.968	0.001
Oral health behaviors	53.34 ± 6.99	53.33 ± 6.63	0.883	54.27 ± 8.20	57.52 ± 6.62	0.001	0.513	0.001
Perceived self-efficacy	43.33 ± 5.95	42.98 ± 5.98	0.705	43.01 ± 6.08	44.85 ± 4.51	0.051	0.770	0.011
Perceived barriers	26.36 ± 9.12	26.24 ± 8.05	0.997	27.13 ± 8.60	30.57 ± 8.52	0.018	0.638	0.007
**Teachers**								
Knowledge	40.54 ± 4.46	40.63 ± 4.36	0.969	40.10 ± 3.11	44.50 ± 4.30	0.002	0.824	0.021
Attitude	33.91 ± 7.54	33.91 ± 7.55	1.000	32.27 ± 7.29	39.91 ± 4.11	0.038	0.611	0.003
Oral health behaviors	54.82 ± 5.58	54.80 ± 5.55	1.000	56.63 ± 6.83	69.36 ± 15.27	0.012	0.502	0.015
Perceived self-efficacy	39.81 ± 7.90	39.80 ± 7.91	1.000	39.72 ± 8.14	42.36 ± 5.74	0.039	0.979	0.034
Perceived barriers	34.63 ± 6.10	34.58 ± 6.14	1.000	35.63 ± 7.43	39.90 ± 8.50	0.012	0.734	0.035
**Students**								
Oral health behaviors	15.59 ± 0.27	15.64 ± 0.28	0.671	14.86 ± 0.27	20.06 ± 0.35	0.040	0.941	0.001

Abbreviation: HSPM, Health Promoting Schools Model. ^a^Mean values were significantly different from those before the intervention (paired-samples *t* tests): *P*<0.05. ^b^Mean values were significantly different from those of the control group after the intervention (ANCOVA): *P*<0.05. ^c^Mean values were significantly different from those of the control group before the intervention (independent-samples t test): *P*<0.05.
